# Surgical Treatment of Clubfoot in Children with Moebius Syndrome

**DOI:** 10.3390/children8040310

**Published:** 2021-04-19

**Authors:** Maurizio De Pellegrin, Lorenzo Marcucci, Lorenzo Brogioni, Giovanni Prati

**Affiliations:** 1 Pediatric Orthopedic and Traumatology Unit, San Raffaele Hospital, 20132 Milan, Italy; lore.marcucci93@gmail.com (L.M.); giovanni.prati.7@gmail.com (G.P.); 2 Department of Orthopedic and Traumatology, San Raffaele Hospital, 20132 Milan, Italy; brogionilorenzo@gmail.com

**Keywords:** clubfoot, children, syndromes, Moebius, relapse, peritalar release

## Abstract

Moebius syndrome (MS) is a rare disease, with paralysis of the VI and VII cranial nerves, frequently associated with clubfoot (CF). The aim of this study was to evaluate surgical treatment of CF in MS, providing its peculiarities. Between 1990 and 2019, we collected data of 11 MS patients with unilateral (*n* = 5) or bilateral (*n* = 6) CF, for a total of 17 feet (9R,8L). Six patients (3M,3F) for a total of 10 feet (6R,4L) were treated elsewhere, performing first surgery at an average age of nine months, and in our hospital for relapse surgery at an average age of 4.5 years (Group 1). Five patients (3M, 2F), for a total of seven feet (3R,4L), were primarily treated in our hospital with a peritalar release according to McKay at an average age of 9.4 months (Group 2). Diméglio score was used to assess CF severity. Three questionnaires were submitted for evaluation of subjective and functional results: American Orthopedics Foot and Ankle Society for Hindfoot (AOFAS), Foot and Ankle Outcome Score (FAOS), and Foot and Ankle Ability Measure (FAAM). Average AOFAS/FAOS/FAMM scores were 82.8, 84.8, and 82.3 for Group 1, and 93.2, 94.7, and 95.1 for Group 2 at an average follow-up of 16.9 and 13.3 years, respectively. The average Diméglio score improved from 15.5 to 4.8 in the long-term follow-up in Group 1 and from 14.6 to 3.8 in Group 2. The comparison between the groups showed better results for AOFAS, FAOS, and FAAM scores for Group 2, particularly for pain, function, and foot alignment and for the post-surgical Diméglio score. CF in MS is more severe and presented a higher relapse rate (58.8%) than idiopathic CF. Peritalar release showed no relapse and better subjective and functional results in the long-term follow-up compared to other surgical techniques

## 1. Introduction

Moebius syndrome (MS) is a rare non-progressive congenital disease, characterized by a complete or partial paralysis of the abducens (CN VI) and facial (CN VII) cranial nerves, either unilateral or bilateral, frequently accompanied by the dysfunction of other cranial nerves and by different types of musculoskeletal malformations [1,2].

The prevalence of MS is estimated to be 1/250,000 live births, with equal incidence in both sexes. Most cases are sporadic, but familial cases, representing about 2% of all affected individuals, have been documented [2]. A peculiar clinical sign of MS patients is the complete or partial absence of facial mimicry; in fact, we speak of “children without smile” who present multiple aesthetic, functional (sucking, pronunciation of some phonemes), and psychosocial problems (inability to communicate emotions, social stigma, marginalization, depression) [3].

In MS, orthopedic concerns are reported in the literature with an incidence of 60%, among which clubfoot (CF) is the most relevant condition, present in 32% of patients against around 1 in 1000 births in the general population. They represent, together with facial and eye problems, the most invalidating aspects for patients [2].

The numerous scientific articles dealing with MS rarely address orthopedic concerns and, in most cases, the experience of a single case is reported. Among more than 750 publications found in PUBMED about the syndrome since 1951, only 11 specifically address orthopedic aspects and only 3 publications discuss specifically about CF. Silvani et al. [4] describe 1 case, whereas Purushothamdas et al. [5] describe 12 cases, yet in a more recent publication, although explicitly referring to MS, it is not clear how many CF have been actually observed in the study, which also includes CF in arthrogryposis [6]. MS has been associated with a variety of orthopedic conditions and, when compared with the general population, patients had an increased incidence of Poland syndrome (15%), scoliosis (14%), and limb deficiencies (30%), as well as a myriad of hand conditions (26%) [7,8].

Lennon (1910) [7] was the first to describe orthopedic aspects, including CF, within the syndrome, without reporting any substantial evidence. Throughout the years, CF has always been associated with MS, and only rarely has it been analyzed separately from other aspects of the syndrome. Recently, Picciolini et al. [1] published a systematic review on MS, declaring that CF incidence in this group of patients is around 40%. In an orthopedic study by McClure et al. [8], 23 individuals with MS underwent a detailed orthopedic examination. A total of 14 out of 23 patients (60%) had at least one CF; 4 of them had unilateral CF and 10 were bilateral. One year later, the same authors again published a 35-year retrospective case series that scanned all the documented MS patients in a pediatric institution since 1980. A total of 18 patients out of 44 (41%) had at least one CF; 13 were bilateral and 5 were unilateral [9].

The aim of our study was to evaluate the result of surgical treatment of CF in MS patients collected in a 29-year period study.

## 2. Materials and Methods

Between 1990 and 2019, 11 MS patients (6M, 5F) with unilateral (*n* = 5) or bilateral (*n* = 6) CF, for a total of 17 feet (9R, 8L), who underwent 1 or more surgical procedure/s, were collected. Patients were divided into 2 groups.

**Figure 1:**
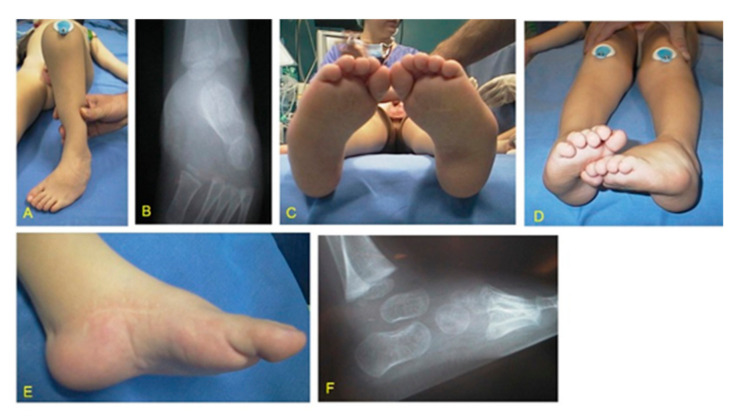
MS (Moebius syndrome) patient with bilateral stiff CF (clubfoot) at 3.5 years of age at the time of relapse surgery. ATL (Achilles tendon lengthening), PR (posterior release), and MR (medial release) were performed at 5 months of age. (**A**) Adduction deformity left; (**B**) X-ray in antero-posterior projection showing pathological talus calcaneal angle; (**C**) plantar view of both feet showing cavus and adduction deformities; (**D**) clinical aspect in frontal plane showing all components of CF; (**E**) medial aspect with skin scars after ATL, PR, and MR; (**F**) X-ray in lateral projection showing pathological talus calcaneal angle.

Group 1. This group included a total of 10 feet (6R, 4L) in 4 patients (2M, 2F) affected by bilateral CF and 2 patients (2M) affected by unilateral CF (2R) who were surgically treated in another institution. The 10 relapsed feet were primarily treated with manipulations and casts in all cases, with medial release (MR) in 8 cases, and with Achilles tendon lengthening (ATL) and posterior release (PR) in all cases (Figure 1). All relapsed feet underwent relapse surgery in our institution at an average age of 4.5 years (range 3.5–7), including ATL in 6 cases, PR in 1 case, posteromedial release in 4 cases, tibialis anterior transfer in 5 cases, cuboid osteotomy in 2 cases, and soft tissue release and Ilizarov external fixator in 1 case.

The mean follow-up period, calculated from the first surgery before relapse surgery, was 2.8 years (range 1–4), while the mean follow-up period after relapse surgery calculated from the first surgery was 16.9 years (range 6.6–24.8). Data of these patients are reported in Table 1. 

**Figure 2:**
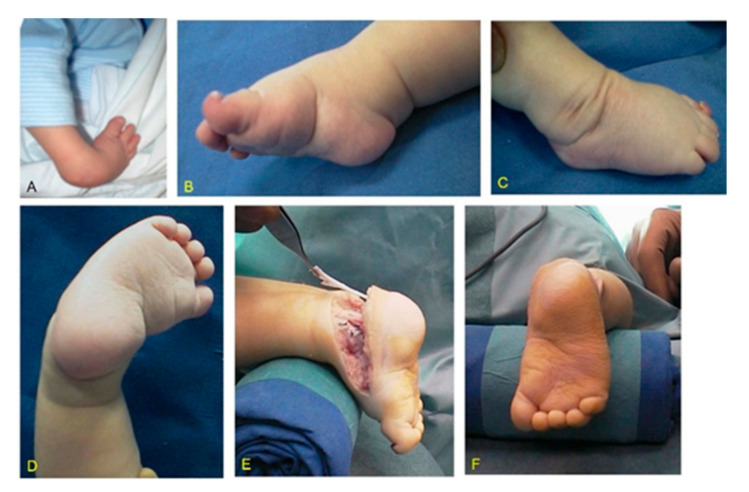
MS (Moebius syndrome) patient with unilateral severe CF (clubfoot) right: (**A**) clinical aspects at birth; (**B**) medial aspect at the time of peritalar release at the age of 7 months showing a rigid equinus, deep crease above the heel, and a transverse crease in the sole; (**C**) lateral aspect with typical skin excess; (**D**) medio-plantar crease; (**E**) postoperative result after peritalar release; (**F**) postoperative plantar aspect.

Group 2. This group included a total of 7 feet (3R, 4L) in 2 patients (1M, 1F) affected by bilateral CF and 3 patients (2M, 1F) affected by unilateral CF (1R, 2L) who were surgically treated in our institution with peritalar release according to McKay [10] at an average age of 9.4 months (range 7–13) and never relapsed (Figure 2).

Data of these patients are reported in Table 2.

The Diméglio score [11] was used to assess CF severity (hence the need for an intervention) of the patients who later underwent surgery in our hospital (either primary or relapse surgery), as well as to objectively evaluate the surgical outcome in the long-term follow-up. The Diméglio score is a clinical evaluation of 4 essential parameters that allow for the classification of CF in 4 grades depending on the stiffness of the deformity. The 4 evaluated parameters are graded from 0 to 4; higher values correspond to an increase in the deformity. The assessment of the foot includes equinus deformity, varus deviation, supination of the foot, and adduction of the forefoot. Four adjunctive parameters, each graded 1 point, are added if pejorative elements exist (posterior and mediotarsal crease, plantar or cavus retraction, and tibialis anterior muscle fibrotic or hypertonic), making the Diméglio score out of 20 points total. Severity is distinguished as follow: Grade I corresponds to “soft–soft feet” with values ranging 0–5, Grade II corresponds to “soft–stiff feet” with values ranging 5–10, Grade III corresponds to “stiff–soft feet” with values ranging 10–15, Grade IV corresponds to “stiff–stiff feet” with values ranging 15–20.

Moreover, patients were asked to fill out 3 questionnaires in order for us to also evaluate the subjective and functional results in the long-term follow-up: (1) American Orthopedics Foot and Ankle Society for Hindfoot (AOFAS) [12], (2) Foot and Ankle Outcome Score (FAOS) [13], and (3) Foot and Ankle Ability Measure (FAAM) [14].

The AOFAS questionnaire is a standard method of reporting clinical status of the ankle and foot. The system incorporates both subjective and objective factors into numerical scales to describe function, alignment, and pain of the ankle–foot complex. AOFAS describes 4 similar questionnaires used to evaluate 4 different anatomical sites of the foot, each graded from 0 to 100. For our purpose, only the ankle–hindfoot grading scale was used because it evaluates the functionality of the ankle, subtalar, talo-navicular, and calcaneo-cuboidal joint levels and can be applied to CF. A grade from 0 to 100 is given: <70 = poor, 70–79 = fair, 80–89 = good, 90–100 = excellent.

The FAOS questionnaire is intended to evaluate symptoms and functional limitations related to the foot and ankle. It consists of a 42-item questionnaire assessing patient-relevant outcomes in 5 separate subscales (pain, other symptoms, rigidity in daily activities, functionality in sports activities, and quality of life). All items are scored from 0 to 4 and each of the 5 subscale scores were calculated as the sum of the items included. The total score of the questionnaire is 168. Raw scores are then transformed in a scale from 0 to 100: <70 = poor, 70–79 = fair, 80–89 = good, 90–100 = excellent.

The FAAM questionnaire is a specific measuring tool for the functional assessment of ankle and foot disorders. It consists of 29 items, divided into 2 categories: daily activities and sport activities. For each item, patients are asked to select the most appropriate among the following options: no difficulty at all, slight difficulty, moderate difficulty, extreme difficulty, unable to do, or non-applicable. Each item receives a score between 0 (unable to do or non-applicable) and 4 (no difficulty at all), and the total score is obtained by the sum of the scores for each item. The maximum score is therefore 116, which is then transformed in a scale from 0 to 100: <70 = poor, 70–79 = fair, 80–89 = good, 90–100 = excellent.

These tests were chosen instead of an X-ray evaluation of the foot [15] because they better meet the patient’s expectations.

A statistical analysis to compare the 2 groups was not reasonable due to the small number of patients, and only descriptive statistics have been performed.

Ethical Approval and Consent to Participate: The study was performed in accordance with the ethical standards in the 1964 Declaration of Helsinki. All authors have read and approved the paper. All authors confirm that they have met the criteria for authorship as established by the International Committee of Medical Journal Editors and believe that the paper represents honest work and are able to verify the validity of the results reported.

## 3. Results

Of 17 surgically treated feet (9R, 8L), belonging to 11 patients (6M, 5F), 10 (6R, 4L) showed relapse while 7 (3R, 4L) did not; thus, 58.8% of CF in MS (clubfoot in Moebius syndrome) relapsing.

**Figure 3:**
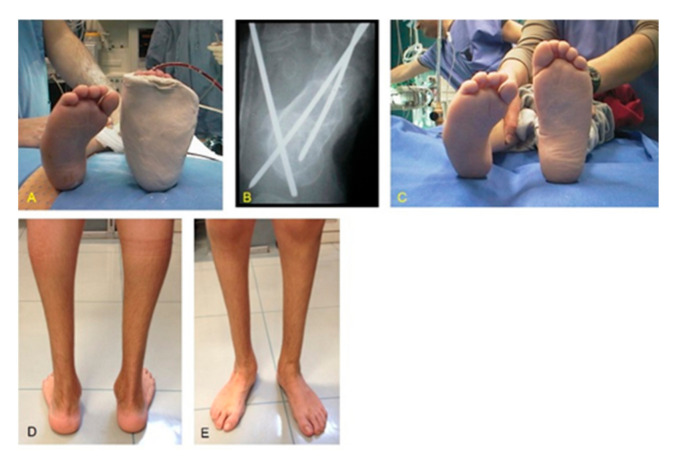
Same patient as shown in Figure 1: (**A**) postoperative cast immobilization after relapse surgery (ATL, PR, PM, tibialis anterior transfer, and cuboid osteotomy); (**B**) X-ray showing correction of the talocalcaneal relationship and K-wires fixation; (**C**) clinical aspect of both feet at the time of relapse surgery of CF right; (**D**) clinical results at a 18.2-year of follow-up with good alignment and correction of the hind foot; (**E**) frontal view showing correction of all CF components.

The functional results according to AOFAS, FAOS, and FAAM scores and Diméglio scores after second surgery of the 10 relapsed CF (Group 1) are reported in Table 3. (Figure 3).

**Figure 4:**
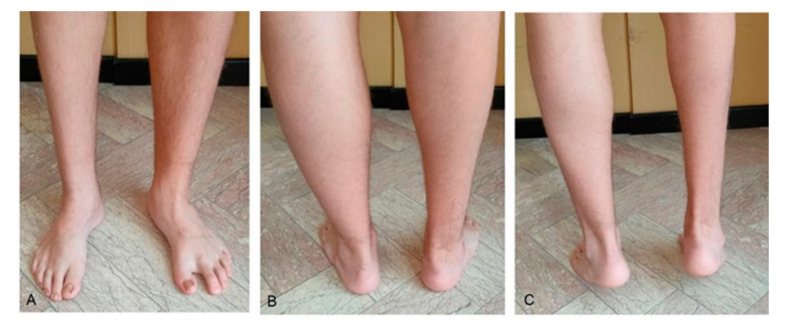
Same patient as shown in Figure 2: clinical aspects at long-term follow-up after 13.7 years. (**A**) Frontal view; (**B**) normal hind foot alignment; (**C**) Achilles tendon evaluation in tiptoes position.

The functional results according to AOFAS, FAOS, and FAAM scores and Diméglio scores after peritalar release of the seven non-relapsed feet (Group 2) are reported in Table 4. (Figure 4).

A comparison of the results between Group 1 and 2 in the long-term follow-up is reported in Table 5. Group 1 received a “good” score (80–89), while Group 2 obtained an “excellent” score (90–100) in all questionnaires.

Regarding the Diméglio score before and after relapse surgery for Group 1 and before and after McKay surgery for Group 2, the former started from an average of 15.5 and ended with an average score of 4.8, while the latter started from an average of 14.6 and ended with an average score of 3.8.

## 4. Discussion

The main worry that afflicts both patients and families is the absence of facial expression, the absence of smile, and all the concerns that derive from it. CF in MS patients represents the second major problem that is subjectively referred and clinically objectionable, with difficulties in walking, running, and practicing sport activities, but also in socializing and wearing normal footwear. From an orthopedic point of view, the most important condition of musculoskeletal malformations in MS is CF. Nevertheless, only three publications have specifically discussed CF [4,5,6]. The series by Purushothamdas et al. [5] reported the outcomes of treatment of 12 neglected CF in seven patients in the setting of MS. Six patients underwent primary posteromedial release. Seven of 12 feet required additional surgery in the form of calcaneal osteotomy in three cases, tibialis anterior transfer in two cases, and extensor hallucis longus transfer in two cases. Guerra-Jasso et al. [6], in a recent publication, discussed CF in arthrogryposis and in MS together; however, precise data about CF in MS are missing.

In the series from McClure et al., 12 out of 18 patients required at least one surgical procedure (not including ATL considered part of Ponseti method), and 7 out of these 12 patients underwent a revision procedure.

Overall, according to the literature [5,6,9], CF in MS seems to be more rigid, more difficult to correct, and has a higher risk of primary unsuccessful results, hence requiring a revision surgery. For the evaluation of the CF in MS, the classification according to Diméglio, used in our study, showed an average combined score of 15.1 (for Group 1 before relapse surgery and for Group 2 before surgery), corresponding to a very severe CF. However, insufficient attention is given in this classification, beyond the deformity itself present on all planes and the posterior and middle plantar folds, to the rigidity of the deformity itself, which appears to be the most peculiar characteristic of CF in MS.

In our study, data regarding 17 surgically treated CF in 11 MS patients, collected in a 29-year period, are reported. This represents the highest number ever published on the topic. Another peculiarity of this study is that most of the CF examined were surgically treated by the same surgeon (M.D.P.) either for primary or relapse surgery. It has emerged from data coming from literature that CF in MS is a complex CF [16]. Ponseti himself considers the CF in MS among the complex and atypical CF; CF characteristics in MS, especially rigidity, represents a potential failure of Ponseti method [17].

Our experience increased both in the treatment of relapses and in the primary treatment of CF in MS according to the surgical procedure described by McKay [10] and Simons [18]. According to these authors, only a restoration of the relationships between talus, navicular, and calcaneus, possible only through an extensive surgery known as peritalar release, guarantees long-term successful results. The technique, widespread until the 1990s, went on to lose its importance because of its excellent results in the short-term but less brilliant results in the long-term, and because of the revival of the Ponseti method [19]. Guerra-Jasso et al. [6] reported the experience with Ponseti method adopted as the first treatment of the CF in MS, although both Guerra-Jasso and Ponseti himself assessed that the failure rate can be higher and the need for further surgical interventions will be likely. For these reasons, adopting a primarily surgical technique for correction of a complex CF such as the one associated with MS was obvious in our department.

The other aspect that significantly conditioned the indication to radical treatment as early as possible was the high number of CF that came to our observation with a serious recurrence after being treated with other surgical methods in other institutions (Group 1) [20]. The severity was also documented with a Diméglio score of 15.5 (very severe) in these relapsed CF, wherein rigidity was again the main feature. Relapse of CF in MS immediately appeared as an important problem of the syndrome.

In these cases, the function; the biomechanical axes of the hindfoot, midfoot, and forefoot; and the impossibility of walking, even with tailored footwear, were seriously compromised, as documented by the Diméglio classification. Even at the expense of functionality, the only possibility of treatment was the restoration of the axes and the maintenance of the correction; the first reachable with posterior release, medial release, and cuboid osteotomy, and the second with tibialis anterior tendon transfer.

The patients primarily treated according to McKay [10] and Simons [18] principles that came to our observation during routinely follow-up showed since the beginning excellent results in the short- and medium-term. In these cases, the CF was classified, before surgery, as “severe” according to Diméglio classification, with an average score of 14.6. The long-term results collected in this study (Table 4) also confirmed the initial trend. No relapse occurred in this group of patients (Group 2), and none of these patients underwent further surgery after peritalar release at an average age of 9.4 months.

According to Turco, Laaveg and Ponseti, and Cooper and Dietz, only a long-term follow-up can be considered in terms of judging the validity of a treatment [21,22,23]; they reported a combined recurrence rate of 42.3% in a long-term follow-up of treatment of idiopathic CF. In our study, relapses in CF in MS were present in 58.8% of cases after primary surgery, higher than the 42.3% relapse rate of idiopathic CF, regardless of the surgery they received (ATL, PR, posteromedial release). However, data reported in the literature about recurrence in idiopathic CF treated with McKay’s procedure were lower, roughly 34.1% [24,25].

In an already rigid CF, such as CF in MS or CF in arthrogryposis, the objective is to correct all components of the deformity with a good alignment of the foot, accepting an eventual stiffness that may result from extensive surgery.

Three questionnaires were chosen instead of one for a better evaluation of the functional clinical result. This is because, beyond the clinical and/or radiographic aspect, general consensus states that the long-lasting functional result is the main goal of CF treatment. The clinical aspect was taken into consideration with the revaluation according to Diméglio, which showed an improvement of the score from an average of 15.5 to 4.8 in Group 1 and from an average of 14.6 to 3.8 in Group 2. These data suggest that a reduction of the number of operations performing an earlier radical surgical procedure can result in potentially better scores.

Functional outcomes are also different in the two groups: results were good for Group 1 and excellent for Group 2. This shows that a single extensive intervention, such as peritalar release, appears more suitable than initial treatments with AL, posterior release, or medial release.

Group 1 patients have in fact undergone multiple surgeries due to recurrence, an aspect not to be underestimated in the management of a syndrome that already needs other surgeries for its other malformations, such as eye and facial surgery (smile surgery).

It should also be noted that the average ages at the time of the first surgical treatment for Group 1 and Group 2 were 9 and 9.4 months, respectively. In idiopathic CF, first surgery usually varies from 2 to 12.4 months (average 5.2) [26] and even earlier in the Ponseti step of ATL intervention (average < 3 months) [19]. This could be explained by the fact that the diagnosis of a syndrome, not always recognized at birth, such as MS, requires overall evaluation of the newborn. The latter can cause a late classification and a late adequate treatment of the complex CF.

There are many limitations in this study. First of all, the two presented groups had no comparable epidemiological data, since Group 1 patients referred to our hospital specifically for relapse surgery after different non-surgical and surgical treatment, while Group 2 patients referred to the same surgeon (MDP) for initial homogenous treatment. Thus, statistical analysis to compare the two groups, as well as the small number of feet, was not possible, and only a retrospective observational study could be conducted. Another limitation regards the high percentage of relapse in the total number of feet observed; as mentioned before, Group 1 consists of patients who specifically came for relapse surgery, and no data are available regarding non-surgical or surgical treatment of CF in MS that did not relapse. However, according to the Italian Moebius Syndrome Association [1], only a small number of CF in MS has an uncomplicated follow-up without relapsing (unpublished data). Nevertheless, despite the small number involved in the study, Group 2 showed no relapse and better subjective and functional results in the long-term follow-up compared to other surgical techniques. Another limitation regards the evaluation of stiffness, which appears to be the principal characteristic of the CF in MS and is underestimated. The stiffness chapter of the FAOS questionnaire uses a subjective judgment in the evaluation by asking questions such as “feeling after wakening” or “after sitting”, which undoubtedly are aimed at an assessment of daily life compared to an objective measurement of ankle/foot mobility, which is often markedly reduced in CF in MS.

In this regard, the analysis of the subchapters scores concerning pain, function, and alignment for the AOFAS questionnaire was useful. Analyzing these results, we found how the function is subjectively satisfactory, albeit in the presence of a reduced range of motion of the ankle and subtalar joint, and how the alignment was less satisfactory in Group 1 compared to Group 2 (Table 3, Table 4 and Table 5).

## 5. Conclusions

Due to its peculiar clinical characteristics, such as severity of the deformity and rigidity, CF in MS can be considered as complex and must be distinguished from idiopathic CF. In the majority of cases, CF in MS, either primary or relapsed, can be classified as “severe” or “very severe” according to the Diméglio classification. CF in MS primarily treated with ATL, PR, and PM showed a high rate of relapse, while CF in MS primarily treated with a peritalar release surgical procedure showed no relapse. Relapse surgery with ATL, PR, MR, tibialis anterior tendon transfer, cuboid osteotomy, and Ilizarov device showed very good clinical and functional results in the long-term follow-up and a clear improvement of Diméglio score. Primary surgical correction with peritalar release showed excellent clinical and functional results in the long-term follow-up and also a clear improved Diméglio score. Peritalar release showed no relapse and better subjective and functional results in the long-term follow-up compared to other surgical techniques.

## Figures and Tables

**Table 1:** Group 1 data of patients with relapsed feet after first surgery who underwent second surgery for correction. Diméglio score is reported before second surgery.

Patient	Sex	Clubfoot Side	Age at 1st Surgery (Months)	Surgery (1st)	FU 1 (Years)	Relapse	Dimeglio Score	Age at 2nd Surgery (Years)	Surgery (2nd)	Relapse	FU 2 (Years)
1	F	Bilateral	4	ATL, PR, MR	3	YES	15 Bilateral	3.5	ATL, PR, MR, TAT	NO	19.7
2	F	Bilateral	8	ATL, PR, MR	2	YES	16 Bilateral	7	ATL, PR, MR, ILZ	NO	7.8
3	M	Bilateral	5	ATL, PR, MR	3	YES	13 Bilateral	3.5	ATL, PR, MR, TAT, CO	NO	18.2
4	M	Bilateral	4	ATL, PR, MR	1	YES	18 Bilateral	4	ATL, PR, MR, TAT, CO	NO	6.6
5	M	R	21	ATL, PR	4	YES	n.e. *	5	ATL, TAT	NO	24.5
6	F	R	12	ATL, PR	4	YES	n.e. *	4	ATL, TAT	NO	24.8
Average			9		2.83		15.5	4.5			16.93

ATL (Achilles tendon lengthening), PR (posterior release), MR (medial release), TAT (tibialis anterior transfer), CO (cuboid osteotomy), ILZ (Ilizarov), FU 1 (follow-up after first surgery), FU 2 (second follow-up after first and relapse surgery), * n.e. (not evaluable).

**Table 2:** Group 2 data of patients who primarily underwent peritalar release for correction. Diméglio score is reported before surgery.

Patient	Sex	Clubfoot Side	Initial Dimeglio Score	Age at Surgery (Months)	Surgery	Relapse	Follow-Up (Years)
1	F	L	14	9	Peritalar release	NO	6.6
2	M	L	13	9	Peritalar release	NO	10.2
3	M	R	16	7	Peritalar release	NO	13.7
4	M	Bilateral	12	13	Peritalar release	NO	16.1
5	F	Bilateral	18	9	Peritalar release	NO	19.9
Average			14.6	9.4			13.3

**Table 3:** Group 1 results of patients with relapsed feet after first surgery who underwent second surgery for correction. AOFAS, FAOS, and FAAM scores with parameters composing the total scores (<70 = poor, 70–79 = fair, 80–89 = good, 90–100 = excellent). For FAOS, the total score of the questionnaire is 168. Raw scores are then transformed into a scale from 0 to 100. For FAMM, the maximum score is 116, which is then transformed in a scale from 0 to 100. Diméglio scores were reported after second surgery. * n.e. = not evaluable.

	AOFAS Score				FAOS Score						FAAM Score			Dimeglio Score at FU
	Pain	Function	Alignment	Total Score (%)	Stiffness	Pain	Daily Activities	Sport Activities	QoL	Total Score (%)	Daily Activities	Sport Activities	Total Score (%)	Total Score (Higher Score Means Worse Result)
Max Score	40	50	10	100	28	36	68	20	16	100	88	28	100	20
1	40	46	10	96	24	34	65	18	14	92.2	87	23	94.8	4
2	30	42	5	77	20	28	59	12	13	78.6	71	15	74.1	5
3	40	46	10	86	24	30	66	16	15	89.9	84	19	88.8	5
4	30	40	5	75	21	28	58	13	14	79.8	67	21	75.9	5
5	40	42	5	87	25	32	63	15	15	89.2	77	22	85.3	n.e. *
6	30	41	5	76	21	26	58	14	14	79.2	69	18	75.0	n.e. *
Average	35	42.8	6.7	82.8	22.5	29.7	61.5	14.7	14.2	84.8	75.8	19.7	82.3	4.8

**Table 4:** Group 2 results of patients who underwent peritalar release. AOFAS, FAOS, and FAAM scores with parameters composing the total scores (<70 = poor, 70–79 = fair, 80–89 = good, 90–100 = excellent) and Diméglio score. For FAOS, the total score of the questionnaire is 168. Raw scores are then transformed onto a scale from 0 to 100. For FAMM, the maximum score is 116, which is then transformed onto a scale from 0 to 100.

	AOFAS Score				FAOS Score						FAAM Score			Dimeglio Score at FU
	Pain	Function	Alignment	Total Score (%)	Stiffness	Pain	Daily Activities	Sport Activities	QoL	Total Score (%)	Daily Activities	Sport Activities	Total Score (%)	Total Score (Higher Score Means Worse Result)
Max Score	40	50	10	100	28	36	68	20	16	100	88	28	100	20
1	40	43	10	93	26	36	67	16	16	95.8	85	25	94.8	3
2	40	45	10	95	28	36	68	17	16	98.2	86	25	95.7	4
3	40	44	10	94	22	35	68	17	15	93.5	86	24	94.8	4
4	40	47	10	97	22	35	66	16	16	92.3	87	26	97.4	3
5	30	47	10	87	27	32	66	17	15	93.5	86	22	93.1	5
Average	38	45.2	10	93.2	25	34.8	67	16.6	15.6	94.7	86	24.4	95.1	3.8

**Table 5:** Comparison of the AOFAS, FAOS, and FAAM scores and Diméglio scores between Group 1 and Group 2 before and after surgery.

	Group 1 (Average)	Group 2 (Average)
AOFAS	82.8	93.2
FAOS	84.8	94.7
FAAM	82.3	95.1
Diméglio before surgery *	15.5	14.6
Diméglio after surgery	4.8	3.8

* in Group 1 before surgery means before relapse surgery.

## Data Availability

The datasets used and/or analyzed during the current study are available from the corresponding author on reasonable request.
